# Effect of Carboxyl Content on Mechanical Properties of Lignin/Carboxylated Nitrile Rubber Compounds

**DOI:** 10.3390/polym17172332

**Published:** 2025-08-28

**Authors:** Hongbing Zheng, Dongmei Yue

**Affiliations:** Key Laboratory of Beijing City on Preparation and Processing of Novel Polymer Materials, Beijing University of Chemical Technology, Beijing 100029, China; zhenghongbing@petrochina.com.cn

**Keywords:** lignin, carboxylation, NBR, compounds

## Abstract

Nitrile rubber (NBR) exhibits excellent oil resistance, wear resistance, gas barrier properties, and mechanical properties. On the other hand, lignin, a by-product of the pulp and paper industry, can serve as an ideal substitute for carbon black as a reinforcing agent for rubber. However, when NBR is directly compounded with lignin, direct compounding fails to achieve the desired reinforcing effect due to poor dispersion of lignin in the NBR matrix and poor compatibility with the NBR phase. In this paper, carboxyl groups were introduced via cyano group hydrolysis. By controlling the hydrolysis time, we successfully prepared two types of carboxylated NBR with different carboxyl contents. Subsequently, the carboxylated NBR was processed into lignin/NBR composites via dry blending. The results indicated that the introduction of carboxyl groups endowed NBR with higher polarity and reactivity, significantly enhancing the interfacial compatibility between lignin and the rubber matrix. The mechanical properties of the composite were greatly improved, with the mechanical strength increasing from 4.5 MPa without carboxyl groups to 13.8 MPa with high carboxyl content. The good dispersion of lignin also significantly improved the thermal stability of the composite. The carboxylation modification strategy of NBR provides a new approach for preparing high-performance NBR/biomass composites.

## 1. Introduction

NBR is a synthetic rubber produced by emulsion copolymerization of butadiene and acrylonitrile [1], exhibiting excellent oil resistance, wear resistance, airtightness, and good physical and mechanical properties [2,3,4]. Due to the presence of nitrile groups in its molecular structure [5], NBR shows strong resistance to non-polar or weakly polar oils, thus being widely used in various fields such as petroleum [6], the chemical industry [7], and the automotive industry [8]. However, NBR also has some drawbacks, including relatively poor cold resistance [9]. Moreover, as the acrylonitrile content increases, the elasticity of the rubber decreases, and its heat-resistant oxidative aging performance still needs to be improved [10]. Carboxylated nitrile rubber (XNBR) is a high-performance rubber prepared by introducing carboxyl functional groups into nitrile rubber [11,12]. The carboxylation process typically involves introducing carboxyl groups onto the molecular chains of nitrile rubber through specific chemical reactions [13]. This process not only changes the chemical structure of the rubber [14] but also significantly affects its physical properties [15]. Compared with conventional nitrile rubber, XNBR has better tensile strength, tear strength, and hardness [16], and its interaction with (other materials) [17] can improve the overall performance of composite materials. However, carboxylated nitrile rubber also faces some problems: due to the strong polarity of carboxyl groups [18], XNBR has a high cohesive energy density, resulting in poor processing properties such as high heat generation and poor fluidity during mixing [19]. In addition, the cost of XNBR is relatively high [20], which limits its large-scale application to some extent. Carbon black is the main reinforcing filler for rubber, but it is mainly derived from fossil fuels. Therefore, the use of other industrial wastes or biomass resources as reinforcing fillers for rubber has become a new development trend. In the field of NBR, Yang et al. [21] reported the use of fly ash (FA) as a reinforcing filler for XNBR. Through the in-situ carboxylate reaction between the carboxyl groups of XNBR and FA particles, a fixed rubber layer was formed on the surface of the FA particles, thus achieving better interfacial adhesion. The tensile strength of the XNBR/20FA composite reached 23.19 MPa, which was about 44.0% higher than that of pure XNBR. Szadkowski et al. [22] reported a modified NBR composite containing hydroxylated multi-walled carbon nanotubes (MWCNT-OH). After modification, the torque, crosslinking density, dynamic modulus, and tensile strength of the NBR vulcanizate were all improved. Martyna Pingot et al. [23] reported the reinforcing effect of cellulose nanofibers (CNF) on NBR, aiming to determine the potential of carbon nanofibers as reinforcing fillers in rubber technology. Ha et al. [24] reported the filling of modified silica (SiO_2_) into XNBR, where the carboxyl groups of XNBR reacted with zinc ion-coated nano-silica fillers to form an ionomer elastomer.

Lignin is the second most abundant biomass resource in nature, second only to cellulose [25], and also the most abundant aromatic biomass compound [26]. Lignin macromolecules are typically composed of syringyl (S), guaiacyl (G), and hydroxyphenyl (H) units [27]. These units are randomly connected through C-C or C-O bonds, forming a highly branched molecular structure [28]. The aromatic units in lignin, combined with strong intramolecular and intermolecular interactions such as hydrogen bonding and π-stacking [29], endow lignin molecules with rigidity, making them potential reinforcing agents for polymer materials [28,30]. In the papermaking and biorefinery industries, lignin is often regarded as a low-value waste [31]. The papermaking industry generates nearly 50 million tons of lignin waste annually, only a small portion of which is utilized [32]. A large amount of lignin is incinerated or discharged in the form of papermaking waste liquor, causing serious pollution. In today’s era of encouraging and supporting sustainable green development [33], the high-value development and utilization of lignin waste has become an inevitable trend towards high-quality green development [34]. Lignin has a long history of application as a rubber reinforcing agent [35]. However, lignin contains a large amount of phenolic hydroxyl/alcoholic hydroxyl groups [36], and its inherent strong hydrogen bonding makes it difficult for lignin to disperse well in rubber matrices [37]. Especially when blended with non-polar rubbers, such as natural rubber, the interfacial compatibility between the two is extremely poor, and the reinforcing effect is very limited [38]. Common methods for reinforcing rubber with lignin include high-temperature hot-melt dynamic treatment and solvent extraction fractionation of lignin. High-temperature hot-melt dynamic treatment can fully mix lignin and rubber, but it requires high energy consumption, and high-temperature treatment can damage the structure of rubber molecular chains. Solvent extraction fractionation of lignin requires a large amount of toxic reagents. Compared with other industrial lignin, lignosulfonate has higher polarity and matches the strongly polar XNBR. The hydrophilicity of its sulfonic acid groups can reduce the cohesion between polar rubber molecular chains, improve the fluidity during the mixing process, and allow it to be directly blended with XNBR through traditional mixing methods. Therefore, it is the best industrial lignin for reinforcing XNBR [19,20].

By combining lignin with XNBR, the issues of poor processability and high cost associated with XNBR can be addressed. The carboxyl group possesses strong reactivity and high polarity [18], which can enhance the interfacial compatibility between lignin and the rubber matrix, ensuring the sufficient dispersion of lignin in the rubber matrix. Furthermore, a chemical reaction may occur between the carboxyl group and the hydroxyl group of lignin, forming a covalent bond, which will further strengthen the interaction between the two materials. In this paper, nitrile rubber is converted into a carboxylated product through a cyanide hydrolysis reaction. By controlling the hydrolysis time, two types of carboxylated nitrile rubbers with different carboxyl contents (0.68–1.13% and 2.48–3.15% carboxyl content, respectively) are successfully prepared. It is worth noting that, unlike the preparation strategy of XNBR/lignin composites reported in the literature [39], this study is the first to synthesize XNBR in situ through a self-developed phase transfer catalytic hydrolysis technique (including an organic acid acidification step). This method has not been reported previously (see Section 2.3 for details). Subsequently, the self-made XNBR is processed into lignin/XNBR composites through a dry blending process, and the effects of carboxyl content on the mechanical properties and thermal stability of the composites are investigated. The results indicate that the introduction of carboxyl groups significantly enhances the interfacial compatibility between lignin and the rubber matrix, with the mechanical strength of the composite increasing from 4.5 MPa (without carboxyl groups) to 13.8 MPa (with high carboxyl content). The good dispersibility of lignin also improves the thermal stability of the composite. The new method for carboxylation modification of NBR established in this study provides an innovative approach for the preparation of high-performance biomass composites.

## 2. Materials and Methods

### 2.1. Materials

NBR N220S, with a bound acrylonitrile content of 41%, produced by Japan’s JSR Corporation; sodium lignosulfonate purchased from Yunnan Yunjing Forest Paper (95%, with a sulfonic acid group content of approximately 1.55 mmol/g, a weight-average molecular weight (*M*_w_) ranging from 20,000 to 40,000, and a particle size range of 10–100 μm as measured by laser particle size analysis); sodium hydroxide (NaOH, 99.0%), tetrabutylammonium bromide (TBAB, 99%), chlorobenzene (99.5%), trifluoroacetic acid (CF_3_COOH, 98.0%), pyridine (99%), zinc oxide (ZnO, 99.9%), stearic acid (SA, 99.0%), tetramethyl thiuram disulfide (TMTD, 99.0%), 2,2′-dibenzothiazole disulfide (DM, 98%), and sulfur (S, 99.5%) are all analytically pure and were purchased from Shanghai Titan Science Co., Ltd., Shanghai, China.

### 2.2. Performance Testing and Characterization

Fourier transform infrared spectroscopy (FT-IR) measurements were conducted using a Thermal Scientific Nicolet iS5 spectrometer (Perkin Elmer Inc., Waltham, MA, USA). The resolution was 4 cm^−1^, and each recorded spectrum was obtained through 32 scans, covering a wavenumber range from 4000 to 500 cm^−1^.

The morphology of lignin/XNBR rubber composites was observed using a Nova Nano SEM 430 scanning electron microscope (FEI, Eindhoven, The Netherlands) at an acceleration voltage of 10 kV. The fractured surfaces of rubber samples for SEM were obtained by segmenting the bulk samples and quenching them in liquid nitrogen. Prior to observation, all samples were coated with a thin layer of gold.

After being stored at room temperature for 24 h, the vulcanized rubber was cut into dumbbell-shaped samples according to the [/T 528-2009 standard [40]], and its thickness was measured. The mechanical properties were tested using a ZQ-990LB electronic tensile testing machine (Zhiqu Precision Instrument Co., Ltd., Dongguan, China) at a speed of 500 mm/min. The test results were the average values of five parallel tests for each sample.

The transition temperature of the composite rubber was determined using a differential scanning calorimeter (DSC, Mettler-Toledo, Schwerzenbach, Switzerland). The test conditions were as follows: under a nitrogen atmosphere, the sample was first heated from 25 to 80 °C to eliminate thermal history, maintained at this temperature for 5 min, then cooled to −80 °C and held for another 5 min, and subsequently reheated to 80 °C. The heating and cooling rates during the entire process were set at 10 K/min.

Using the DMA 242D dynamic mechanical analyzer from NETZSCH Gerätebau GmbH, Selb, Company, dynamic mechanical analysis (DMA) spectra of rubber composites were obtained. The samples were measured in tensile mode at a constant frequency of 1 Hz, with a temperature range from −100 to 100 °C and a heating rate of 3 °C/min.

Thermogravimetric analysis (TGA) was conducted using the STA 6000 (Perkin Elmer, Inc., Norwalk, CT, USA) in a nitrogen atmosphere within a temperature range of 30 to 800 °C, at a heating rate of 10 °C/min.

### 2.3. Synthesis of Carboxylated NBR (XNBR)

**Figure 1:**
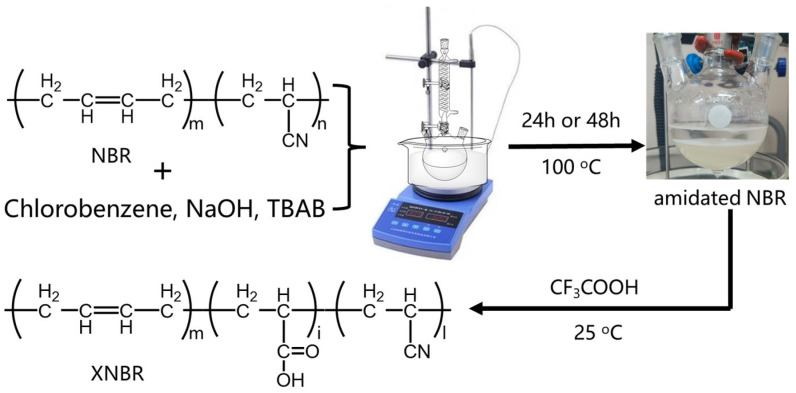
Carboxylation modification process of NBR.

A mixture of NaOH aqueous solution (1.0 M, 20.0 mL) and chlorobenzene (20.0 mL) was charged with NBR (1.0 g, 17.0 mmol, 1.0 equiv.) and tetrabutylammonium bromide (0.3 g, 0.1 M as final concentration). The reaction mixture was vigorously stirred at 100.0 °C for controlled durations to achieve different carboxylation degrees: low-degree carboxylated NBR (24 h) and high-degree carboxylated NBR (48 h). After cooling to room temperature, the aqueous phase was acidified to pH 1.0 with trifluoroacetic acid and extracted with chloroform (3 × 80 mL). The combined organic layers were washed with brine (100.0 mL), dried over anhydrous MgSO_4_, filtered, and concentrated under reduced pressure using a rotary evaporator (Figure 1). The crude product was dissolved in chloroform (10 mL) and precipitated by dropwise addition into vigorously stirred methanol (100 mL). The resulting solid was collected by filtration, washed with fresh methanol (3 × 20 mL), and dried under vacuum at 40 °C for 12 h to afford the title compound as a pale-yellow solid (0.7 g, 70% yield). Significant variations in carboxyl content were observed between 24 h and 48 h hydrolysis batches. Therefore, ten independent syntheses were conducted under identical conditions for each hydrolysis duration. The carboxyl content of all batches was determined according to the analytical method described in Section 2.4. The mass fraction of carboxyl groups in low carboxyl content XNBR obtained after 24 h of hydrolysis is approximately 0.68–1.13% (named IX-NBR); in high carboxyl content XNBR obtained after 48 h of hydrolysis, the mass fraction of carboxyl groups is approximately 2.48–3.15% (named HX-NBR).

### 2.4. Determination of Carboxyl Content in XNBR

(1) Sample Dissolution: Approximately 0.20 ± 0.02 g of XNBR sample was placed into a 250 mL stoppered conical flask containing a magnetic stir bar. 30 mL of pyridine was added using a measuring cylinder. The flask was placed on a magnetic stirrer, and the mixture was stirred gently for at least 5 h until complete dissolution was achieved.

(2) Titration: To the dissolved solution, 6–7 drops of thymolphthalein indicator solution were added (Thymolphthalein Indicator Solution (10 g/L): Accurately weigh 1.0 g of thymolphthalein and dissolve it in 95% ethanol. Dilute the solution to a final volume of 100 mL with an additional 95% ethanol.) The solution was then titrated with standardized 0.05 mol/L potassium hydroxide in ethanol (KOH-ethanol) solution until a stable blue color appeared. The solution was allowed to stand for over 10 min. If the color faded to green or yellow within this period, titration was continued dropwise until the blue color reappeared and persisted unchanged for a further 10 min. The volume of KOH-ethanol titrant consumed was recorded as *V* (mL). A blank titration (without XNBR sample) was performed concurrently following the same procedure, and the volume consumed was recorded as *V*_0_ (mL).

(3) Calculation: The carboxyl group content (*X*, expressed as mass percentage, wt%) in the XNBR was calculated using the following equation:

*X* = [(*V* − *V*_0_) × *c* × 45.03 × 10^−3^]/*m* × 100%

*c* = Concentration of the KOH-ethanol standard titrant (mol/L)

*V* = Volume of KOH-ethanol titrant consumed by the sample (mL)

*V*_0_ = Volume of KOH-ethanol titrant consumed by the blank (mL)

45.03 = Molar mass of the carboxyl group (-COOH) (g/mol)

*m* = Mass of the XNBR sample (g)

10^−3^ = Conversion factor from mL to L (to match concentration units)

The measurement results are presented as the average of five measurements.

### 2.5. Mixing and Vulcanization of XNBR/Lignin Compounds

Basic formula (parts by mass/phr): XNBR 100.0, ZnO 5.0, S 1.5, SA 1.0, DM 1.0, TMTD 0.2, sodium lignosulfonate (10.0, 20.0, 30.0, 40.0).

Mixing process: At room temperature, activate the tap water cooling system of the double-roll mill (X(S)K-160, Wuxi First Rubber and Plastic Machinery Co., Ltd., Wuxi, China). First, plasticize the raw rubber by wrapping it around the rollers and flipping it for 1 min. Then, gradually add sulfur, zinc oxide, and stearic acid. Add the lignin in three batches alternately, flipping left and right alternately, while flipping the rubber and wrapping/rolling it in a triangular shape multiple times until all powders are fully mixed. Finally, add DM/TMTD and continue mixing until evenly distributed. The mixing time for each rubber sample is fixed at 15 min. After all rubber samples are mixed, increase the roller gap, pass each sample through the mill 10 times thinly, and then discharge it. Using the same formula and following the aforementioned procedure, three types of nitrile butadiene rubber (NBR, IX-NBR, HXNBR) with different carboxyl contents were compounded with lignin at different loadings (0, 10, 20, 30, and 40 parts), as detailed in Table 1.

Vulcanization conditions: The rubber compound was left to cure at room temperature for 24 h. Subsequently, a 5.0 g sample of the rubber compound was cut into a circular cake shape, and the vulcanization curve was measured using a rubber processing analyzer (Premier RPA, Alpha Technology Co., Ltd., Hudson, OH, USA) at 165 °C, thereby obtaining torque, scorch time (t10), optimal cure time (t90), maximum torque (MH), and minimum torque (ML). 45.0 g of mixed rubber was placed in a mold and vulcanized using a fully automatic rapid hot press molding machine (KSH-R-149 100T, Dongguan Kesun Industrial Co., Ltd., Dongguan, China) at 165 °C and 20 MPa, according to the corresponding vulcanization time (t90) for the XNBR/lignin composite rubber.

## 3. Results and Discussion

### 3.1. FT-IR Characterization of Carboxylated NBR

**Figure 2:**
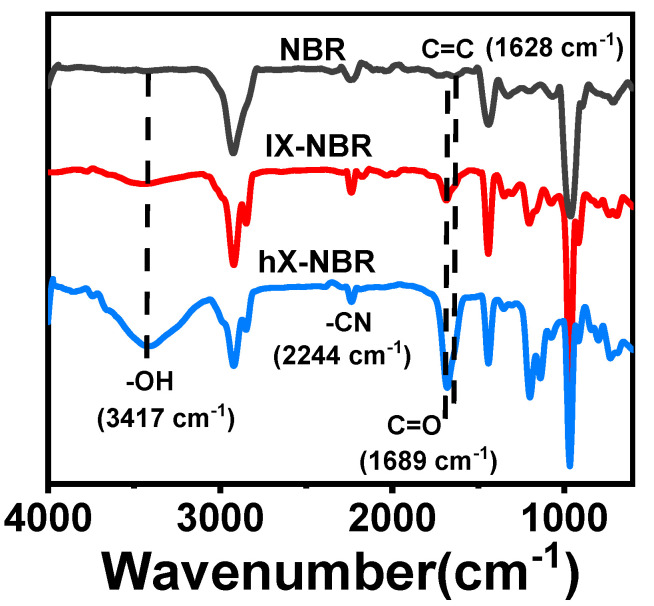
FT-IR spectrum of carboxylated NBR.

Fourier transform infrared spectroscopy (FT-IR) analysis was conducted on XNBR generated through reactions at different times (Figure 2). The stretching vibration peak of carbon-carbon double bonds in the XNBR molecular chain is located at 1436 cm^−1^ [41]; the stretching vibration peak of the cyano group is located at 2236 cm^−1^ [42]. After the carboxylation reaction, two new peaks appeared at 1671 cm^−1^ and 3426 cm^−1^, corresponding to the stretching vibration peaks of carbon-oxygen double bonds and hydroxyl groups in carboxyl groups, respectively [43]. However, the stretching vibration peak of the cyano group at 2236 cm^−1^ still exists. This indicates that some of the cyano groups in NBR have been successfully hydrolyzed to carboxyl groups. In addition, as the reaction time increases, the intensities of the two peaks at 1671 cm^−1^ and 3426 cm^−1^ become stronger, indicating a higher carboxyl content. The carboxyl content in XNBR with different reaction times was determined by titration [44]. The results showed that the mass fraction of carboxyl groups in the sample obtained after 24 h of hydrolysis was approximately 0.68–1.13%; the mass fraction of carboxyl groups in the sample obtained after 48 h of hydrolysis was approximately 2.48–3.15%.

### 3.2. Characterization of Vulcanization Properties of XNBR/Lignin Compounds

**Figure 3:**
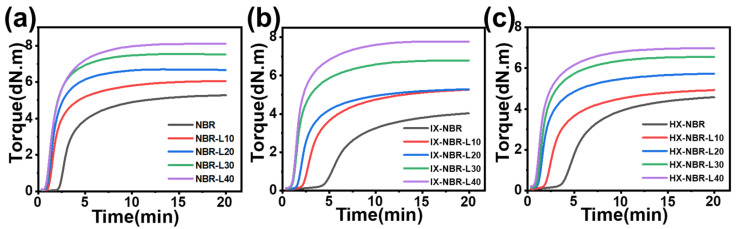
Vulcanization curve diagram of (**a**) NBR-L, (**b**) IX-NBR-L, and (**c**) HX-NBR-L.

By comparing the vulcanization curves of three groups of composites with different carboxyl contents, it can be observed that the addition of lignin significantly promotes the vulcanization process (Figure 3, Table 2). As the lignin content increases, the scorch time and optimal vulcanization time of the composites are significantly shortened, and the vulcanization reaction rate rapidly increases, enabling the composites to achieve a good, vulcanized state. Meanwhile, lignin has a certain reinforcing effect on the composites; as the lignin content increases, the fluidity of the composites decreases. Sodium lignosulfonate contains a large number of polar groups, including sulfonic acid groups (-SO_3_Na), phenolic hydroxyl groups (-ArOH) and their sodium salts (-ArO^−^Na^+^), and methoxy groups (-OCH_3_). These polar groups can form interfacial bonding with the nitrile groups (-CN) in nitrile butadiene rubber (NBR) through dipole-dipole interactions, hydrogen bonding, or ionic bonding. This interaction reduces the viscosity of NBR and improves the dispersibility of vulcanizing agents (such as sulfur, accelerators, zinc oxide, etc.) in the rubber. In addition, the sulfonic acid groups (-SO_3_Na) and phenolic hydroxyl sodium salts (-ArO^−^Na^+^) in sodium lignosulfonate can chemically react with zinc oxide (ZnO) to form zinc sulfonic acid (-SO_3_Zn^+^) and phenolic zinc (-ArOZn^+^). These zinc salt species can enhance the activation efficiency of zinc oxide on accelerators and accelerate the decomposition of accelerators into crosslinking active species (such as thiolate salts), thereby shortening the induction period (scorch stage) of the vulcanization reaction and accelerating the formation rate of crosslink bonds (reducing the vulcanization time) [45].

Furthermore, when comparing NBR, IX-NBR, and HX-NBR under the same formulation conditions, the vulcanization time of nitrile rubber first increases and then decreases with the increase in carboxyl content, which is related to the inhibitory effect of carboxyl groups on the vulcanization process [44]. When the carboxyl content in XNBR is low, the carboxyl groups exhibit a large steric hindrance, hindering the approach of rubber molecular chains, making it difficult to form cross-linking points, thereby prolonging the vulcanization time. As the carboxyl content further increases, the carboxyl groups can undergo acid-base reactions with zinc oxide (ZnO), consuming the zinc oxide in the system. In addition, carboxyl groups can even react with accelerators DM and TMTD, occupying the original reaction sites, leading to the loss of sustained reaction ability in the vulcanization system and appropriately shortening the vulcanization time. This phenomenon affects the cross-linking density and hardness of the composite rubber.

The variation in the difference between the maximum torque (MH) and minimum torque (ML) in the vulcanization curves of IX-NBR and HX-NBR indicates that in the absence of lignin, the crosslinking density initially decreases and then increases with the increase in carboxyl content. However, as the lignin content increases, the crosslinking density of compounds with high carboxyl content gradually decreases, suggesting that lignin has a greater impact on the vulcanization process of XNBR than carboxyl content does on the compounds.

### 3.3. Morphological Characterization of XNBR/Lignin Compounds

**Figure 4:**
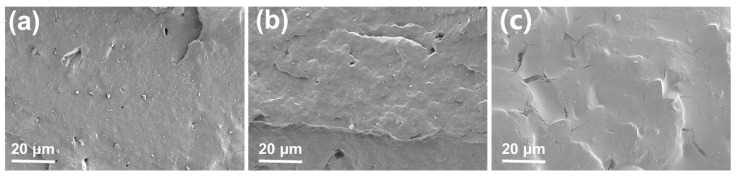
SEM images of (**a**) NBR-L20, (**b**) IX-NBR-L20, and (**c**) HX-NBR-L20.

The scanning electron microscopy (SEM) test results of the fracture surfaces of lignin/NBR composites with different carboxyl contents (lignin content of 40 parts) after liquid nitrogen brittle fracture treatment are shown in Figure 4. ZnO has excellent compatibility with XNBR and can be uniformly dispersed in XNBR, with no obvious granularity observed at a scale of 20 microns. However, the particles in the SEM images have irregular morphologies, appearing as lumps or fragments, thus confirming that they are lignin. For the composite without carboxylation modification (Figure 4a), a large number of lignin particles were observed on the surface of the composite. These particles were not bound to the rubber matrix but were independently distributed on the rubber surface, indicating poor interfacial interaction between lignin and the NBR matrix, as well as poor interfacial adhesion and compatibility between lignin and NBR. After carboxylation treatment, this phenomenon improved (Figure 4b). However, when the carboxyl content was low, some small lignin particles could still be observed on the surface. When the carboxyl content is high (Figure 4c), lignin is tightly bound to the rubber matrix, indicating that carboxylation significantly enhances interfacial compatibility. This improvement stems from a dual mechanism: the increase in carboxyl content enhances the density of the hydrogen bonding network (O-H···O=C) between XNBR and lignin phenolic hydroxyl groups, effectively inhibiting lignin agglomeration; simultaneously, the carboxyl-rich environment promotes esterification reactions (-COOH + HO-lignin→-COO-ester bond) during high-temperature mixing, forming a covalent interfacial layer [39]. These strong interfacial interactions enable the rubber matrix to effectively transfer stress to the lignin phase, while micron-sized lignin particles hinder the sliding of molecular chains through a pinning effect, which is the fundamental mechanism behind the increase in mechanical strength to 13.8 MPa [45].

### 3.4. Mechanical Property Characterization of XNBR/Lignin Compounds

**Figure 5:**
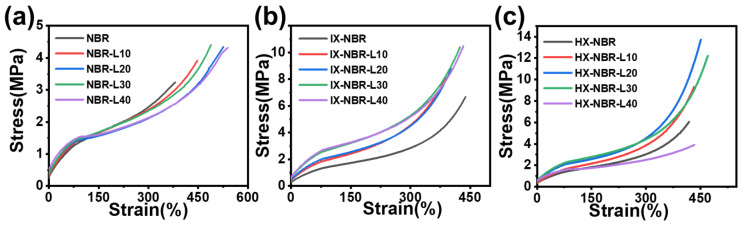
Stress-strain curves of (**a**) NBR-L, (**b**) IX-N BR-L, and (**c**) HX-NBR-L.

**Figure 6:**
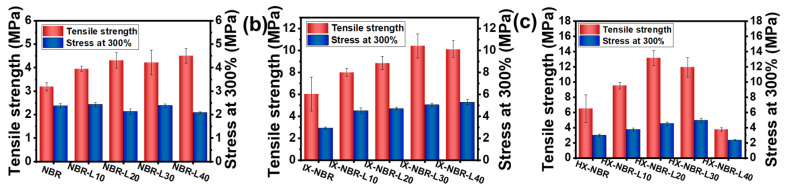
Maximum stress and 300% modulus at a fixed extension of (**a**) NBR-L, (**b**) IX-NBR-L, and (**c**) HX-NBR-L.

The tensile stress-strain curves of lignin with three carboxyl-containing compounds are presented in Figure 5. Figure 6 illustrates the variation trends of tensile strength and 300% modulus for the three carboxyl-containing compounds. For the NBR/lignin composite rubber, the tensile strength exhibits only a slight increase from 3.3 ± 0.2 MPa for neat NBR to 4.3 ± 0.3 MPa for NBR-L40, showing no significant enhancement (Table 3). After carboxylation modification, the tensile strength of the composite rubber increases significantly with the increase in lignin content, rising from 6.7 ± 1.5 MPa for IX-NBR to 10.5 ± 0.8 MPa for IX-NBR-L40, demonstrating an obvious reinforcing effect. This is mainly attributed to the introduction of carboxyl groups, which significantly increases the polarity of NBR molecules, thereby improving their compatibility with the polar lignin reinforcing agent. The two components achieve better miscibility, restricting the slippage of rubber molecular chains. When the carboxyl content is further increased, the tensile strength of the composite rubber shows a trend of first increasing and then decreasing with the increase in lignin content, reaching a maximum of 13.8 ± 1 MPa for HX-NBR-L20. Under the shearing action of the open mill, lignin is more uniformly dispersed in the rubber matrix with a smaller particle size. Numerous active functional groups on the lignin surface can also form hydrogen bonds with carboxyl groups [46], leading to physical crosslinking or even chemical crosslinking [47]. When the composite material is subjected to external tension, the multiple crosslinking networks can prevent the sliding of molecular chains [48], thereby enhancing the mechanical properties, and this effect is strengthened with the increase in carboxyl content. However, when the carboxyl content is excessively high, the mechanical properties of the composite material exhibit a trend of initial increase followed by a decrease as the lignin content increases. Especially when both high carboxyl content and high lignin addition coexist, the tensile strength of the composite rubber is only 3.9 MPa. On the one hand, a large amount of lignin aggregates within the rubber matrix weakens the interaction between the two. In the SEM image (Figure S1), compared to NBR-L40 and IX-NBR-L40, HX-NBR-L40 exhibits a significant presence of cracks and lignin aggregates, undoubtedly having a severe impact on the mechanical properties of the composite rubber. On the other hand, carboxyl groups on XNBR may consume activators in the vulcanization system, such as zinc oxide (ZnO) and molybdenum disulfide (DM), leading to reduced vulcanization efficiency and decreased crosslinking density. These results indicate that an appropriate amount of lignin can form a multi-network crosslinked structure with the NBR matrix, thereby enhancing the mechanical properties of NBR. Excess lignin may distribute unevenly and aggregate, impairing the interfacial compatibility between lignin and the rubber matrix and reducing the mechanical properties of the composite rubber [49].

### 3.5. Strain Scanning Characterization of NBR/Lignin Compounds

**Figure 7:**
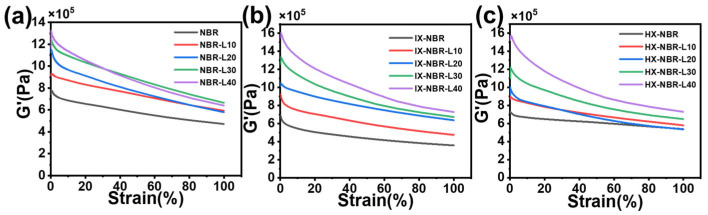
Strain scanning curves of (**a**) NBR-L, (**b**) IX-NBR-L and (**c**) HX-NBR-L.

The Rubber Process Analyzer (RPA) is utilized for constant frequency and constant temperature strain scanning to measure the dynamic properties of NBR/lignin composites under varying lignin loadings and carboxylic acid contents. The Payne effect analysis is utilized to investigate the interaction between fillers in rubber composites [50]. A larger change in storage modulus (ΔG′) indicates a stronger interaction between fillers in the rubber matrix, where ΔG′ is defined as the difference between the storage modulus at the beginning of the test (G′[0]) and the storage modulus at the end of the test (G′[∞]). As shown in Figure 7, in three composite rubbers with different carboxyl contents, the shear modulus (G′) increases with the increase in lignin content. This may be due to the network constraints on rubber chain migration [51], thereby enhancing the crosslinking of rubber composites during vulcanization. Meanwhile, in all three composite rubbers with different carboxyl contents, ΔG’ significantly increases with the increase in lignin content, indicating that lignin forms aggregates mainly caused by hydrogen bonding in the natural rubber matrix, leading to the formation of a filler network in the rubber matrix. Compared to NBR/lignin and IH-NBR/lignin, ΔG′ in XH-NBR/lignin composite rubber is relatively lower, especially when the lignin addition amount is 10 parts and 20 parts. This indicates that, under the premise of a certain lignin addition amount, as the carboxyl content increases, the compatibility between lignin and XNBR improves, lignin aggregation decreases, and mechanical properties enhance. When the lignin addition amount further increases, ΔG′ significantly increases, and lignin begins to aggregate significantly, leading to a decrease in mechanical properties.

### 3.6. Dynamic Thermomechanical Characterization of XNBR/Lignin Compounds

**Figure 8:**
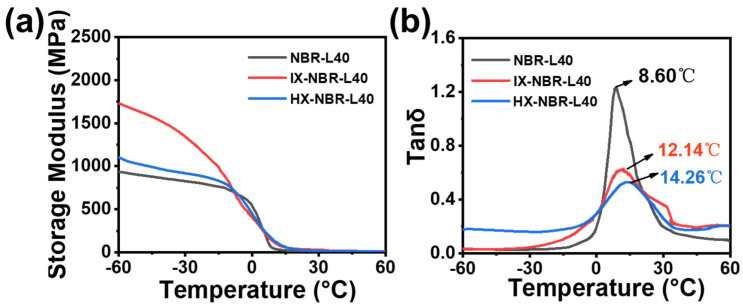
(**a**) Storage modulus and (**b**) Tan δ curves of XNBR/lignin compounds with three different carboxyl contents.

Dynamic mechanical analysis (DMA) was conducted on three XNBR/lignin compounds with different carboxyl contents, all of which contained the same amount of lignin (40 parts). As shown in Figure 8a, with the increase in carboxyl content, the initial storage modulus of the lignin/NBR composites exhibited a trend of first increasing and then decreasing. In the low temperature range from −60.0 to −10.0 °C, the composites without carboxyl groups (NBR) and the HX-NBR showed better stability. After −10.0 °C, the storage modulus of lX-NBR-L40 first decreased sharply and then increased, which is related to the polarity brought by carboxyl groups and the dispersibility of lignin in NBR. As shown in Figure 8b, in the loss factor (Tan *δ*) curves of the three composites, with the increase in carboxyl content, the loss peaks of the three composites showed a significant downward trend, and the glass transition temperature increased from 8.6 °C for NBR-L40 to 14.3 °C for HX-NBR-L40. This indicates that after carboxylation modification, the interaction between lignin and the rubber matrix changed, with lignin better dispersed into the rubber matrix, undergoing closer physical interactions or even chemical crosslinking with the rubber matrix, forming a more stable crosslinked network. As the temperature increases, at the same stretching frequency, the sliding of rubber molecular chains is hindered, thereby improving the dynamic mechanical properties of the composite rubber. By analyzing the Tan *δ* curve obtained from DMA tests, the wet slip resistance of the composite rubber was also evaluated. Generally, the wet slip resistance of rubber is evaluated by the Tan *δ* value at 0 °C, with a higher Tan *δ* value indicating better wet slip resistance. It can be observed that the Tan *δ* value of carboxylated modified composite rubber at 0 °C significantly increases, indicating an improvement in its wet slip resistance [52].

### 3.7. Characterization of Thermal Stability of XNBR/Lignin Compounds

**Figure 9:**
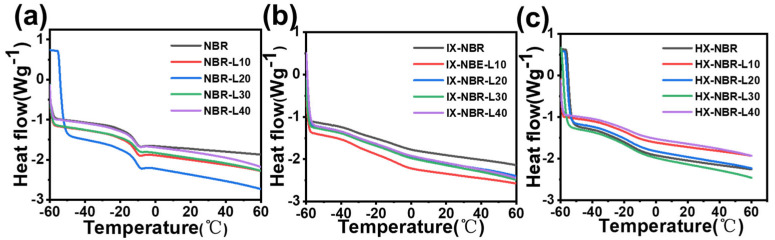
DSC curves of (**a**) NBR-L, (**b**) IX-NBR-L, and (**c**) HX-NBR-L.

The thermal properties of the composite materials were characterized using differential scanning calorimetry (DSC). As shown in Figure 9, in composite rubbers with a lower carboxylic acid content, the glass transition temperature (*T*_g_) of the composite rubber exhibited a trend of first increasing and then decreasing. When the lignin addition level was 20 parts, *T*_g_ reached its maximum value of −7.3 °C. As the lignin content increased to 40 parts, *T*_g_ decreased to −10.0 °C, which was the same as that of NBR without lignin. The *T*_g_ of the composite rubber is primarily determined by the content of lignin with a rigid structure [48] and butadiene with flexible segments [53]. This change is attributed not only to the high flexibility of linear side chains but also to the suppression of intermolecular interactions (hydrogen bonding and π-electron interactions) between lignin molecules. At the same lignin content, as the carboxyl content increases, the *T*_g_ of the composite rubber increases, which is consistent with the behavior observed in DMA.

**Figure 10:**
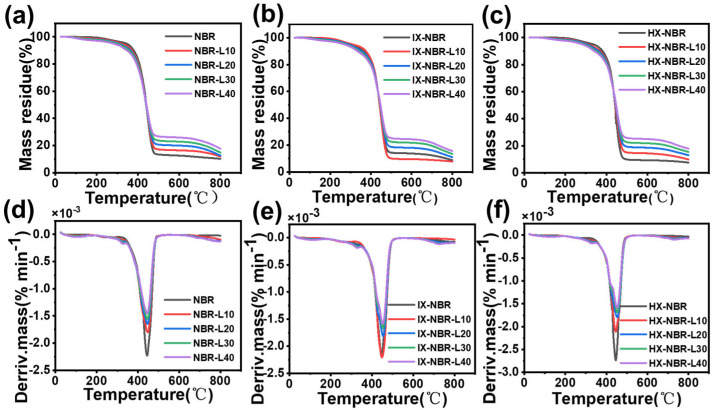
(**a**–**c**) TGA (**d**–**f**) DTG thermograms of XNBR/lignin compounds with three different carboxyl contents.

The thermal stability of the composites was investigated using thermogravimetric analysis (TGA), and the measured thermogravimetric (TG) and differential thermogravimetric (DTG) curves are presented in Figure 10. T05% refers to the temperature at which the sample loses 5% of its mass, which can sensitively reflect the early thermal degradation behavior of the material. T50% is the temperature corresponding to 50% mass loss of the sample; compared with T05%, T50% is more representative of the “core stability” of the material during thermal degradation. As shown in Table 4, for NBR samples without lignin, the T05% values vary with increasing carboxyl content: decreasing from 345.3 °C for neat NBR to 302.5 °C for IX-NBR, and then increasing to 335.1 °C. This phenomenon can be explained by the fact that the thermal stability of cyano groups (-CN) is superior to that of carboxyl groups (-COOH). However, when the carboxyl content is low, partial carboxyl groups start to undergo thermal decomposition. With the further increase in carboxyl content, the carboxyl groups can coordinate with Zn^2+^ ions, thereby alleviating the thermal decomposition. Nevertheless, the T50% values of NBR samples with different carboxyl contents show no significant changes, which may be attributed to the relatively low carboxyl content in XNBR used in this study, resulting in no obvious difference in the core thermal stability represented by T50%. After the addition of lignin, the T05% values of all composite rubbers decrease with increasing lignin content, which may be caused by ash or other impurities in lignin. In contrast, the T50% values of carbonylated NBR composite rubbers all increase, indicating that the incorporation of lignin enhances the degradation resistance of the rubber. This is attributed to the antioxidant effect of lignin, whose phenolic hydroxyl polar groups can inhibit the oxidation of the composites [54]. Additionally, the thermal stability improves with the increase in carboxyl content, as the introduction of carboxyl groups enhances the compatibility between lignin and the rubber matrix. Lignin itself has good thermal stability, and the more uniformly it is dispersed in the rubber matrix, the more stable the cross-linked network formed, thereby improving the overall thermal stability of the composites. The residual mass after TGA (at 600.0 °C) increases with the increase in lignin content, which is due to the high carbon content in lignin.

## 4. Conclusions

In summary, NBR was modified into two types of crosslinked nitrile rubber with different carboxyl contents, and the effects of carboxyl content and lignin addition on the reinforcement performance of NBR/lignin composite rubber materials were investigated through dry blending. The results showed that after carboxylation modification, the interfacial compatibility between lignin and the rubber matrix was improved. Compared to NBR, lignin had a significant mechanical reinforcement effect on XNBR. The maximum tensile strength of NBR/lignin composite materials was only 4.5 MPa (containing 20 parts of lignin). With the same lignin loading, the tensile strengths of low carboxyl content XNBR/lignin composite materials (IX-NBR) and high carboxyl content XNBR/lignin composite materials (HX-NBR) were 8.8 MPa and 13.8 MPa, respectively, representing an increase of 195.6% and 306.7%. When the carboxyl content was low, the mechanical properties increased with the increase in lignin content. In the case of high carboxyl content, the mechanical properties showed a trend of first increasing and then decreasing with the increase in lignin content. Furthermore, after carboxylation modification, lignin had a more significant effect on improving the thermal stability of NBR. Before carboxylation modification, the T50% of the composite rubber only slightly increased from 440.3 to 441.2 °C, showing no significant improvement. After carboxylation modification, especially in composite rubber with high carboxyl content, T50% increased from 442.3 °C to 447.7 °C, thereby better improving the thermal stability of the composite rubber.

## Figures and Tables

**Table 1:** Presents the formulations of NBR, IX-NBR, HXNBR, and lignin, expressed in parts per hundred rubber (phr).

Compound Code	NBR (phr)	IX-NBR (phr)	HX-NBR (phr)	Lignin (phr)
NBR	100	-	-	0
NBR-L10	100	-	-	10
NBR-L20	100	-	-	20
NBR-L30	100	-	-	30
NBR-L40	100	-	-	40
IX-NBR	-	100	-	0
IX-NBR-L10	-	100	-	20
IX-NBR-L20	-	100	-	30
IX-NBR-L30	-	100	-	40
IX-NBR-L40	-	100	-	50
HX-NBR	-	-	100	0
HX-NBR-L10	-	-	100	10
HX-NBR-L20	-	-	100	20
HX-NBR-L30	-	-	100	30
HX-NBR-L40	-	-	100	40

**Table 2:** The curing characteristic parameters of XNBR/lignin compounds.

Samples	t10 (min)	t90 (min)	ML (dN·m)	MH (dN·m)	ΔM (MH-ML) (dN·m)
NBR	2.4	8.6	0.04	5.21	5.17
NBR-L10	1.2	6.5	0.04	6.05	6.01
NBR-L20	1.1	4.5	0.06	6.69	6.63
NBR-L30	0.9	4.4	0.04	7.54	7.50
NBR-L40	0.8	5.2	0.09	8.1	8.01
IX-NBR	4.6	13.1	0.04	4.03	3.99
IX-NBR-L10	2.7	9.9	0.05	5.27	5.22
IX-NBR-L20	2.2	8.1	0.09	5.29	5.20
IX-NBR-L30	1.3	5.8	0.11	6.79	6.68
IX-NBR-L40	1.2	5.7	0.13	7.78	7.65
HX-NBR	3.8	11.5	0.03	4.45	4.42
HX-NBR-L10	2.1	9.3	0.07	4.93	4.86
HX-NBR-L20	1.3	6.8	0.13	5.73	5.60
HX-NBR-L30	1.1	5.8	0.14	6.55	6.41
HX-NBR-L40	1.0	5.6	0.20	6.98	6.78

t90 refers to the optimal vulcanization time (min), t10 refers to the scorch time (min), ML refers to the minimum torque (dN·m), MH refers to the maximum torque (dN·m), and ΔM refers to the difference between the maximum torque and the minimum torque (dN·m).

**Table 3:** Mechanical properties parameters of XNBR/lignin compounds with three different carboxyl contents.

Samples	Tensile Strength (MPa)	Elongation at Break (%)	Stress at 100% (MPa)	Stress at 300% (MPa)
NBR	3.3 ± 0.2	392.1 ± 43.6	1.4 ± 0.1	2.3 ± 0.1
NBR-L10	3.9 ± 0.1	498.9 ± 31.0	1.4 ± 0.1	2.4 ± 0.1
NBR-L20	4.5 ± 0.3	524.6 ± 46.8	1.5 ± 0.1	2.2 ± 0.1
NBR-L30	4.4 ± 0.5	488.4 ± 26.9	1.4 ± 0.1	2.6 ± 0.1
NBR-L40	4.3 ± 0.3	541.0 ± 9.8	1.4 ± 0.1	2.1 ± 0.1
IX-NBR	6.7 ± 1.5	439.3 ± 43	1.4 ± 0.1	2.8 ± 0.1
IX-NBR-L10	8.1 ± 0.4	389.0 ± 11	2.0 ± 0.1	4.6 ± 0.3
IX-NBR-L20	8.8 ± 0.6	402.7 ± 13.0	2.1 ± 0.1	4.8 ± 0.1
IX-NBR-L30	10.4 ± 1.1	427.2 ± 17.0	2.8 ± 0.1	5.2 ± 0.1
IX-NBR-L40	10.5 ± 0.8	432.9 ± 11.7	2.9 ± 0.1	5.7 ± 0.2
HX-NBR	6.1 ± 1.81	418.8 ± 28.0	1.6 ± 0.1	3.0 ± 0.2
HX-NBR-L10	9.3 ± 0.45	432.4 ± 24.0	1.8 ± 0.1	3.9 ± 0.2
HX-NBR-L20	13.8 ± 1	451.3 ± 10.4	2.2 ± 0.1	4.5 ± 0.2
HX-NBR-L30	12.2 ± 1.3	470.7 ± 22.7	2.6 ± 0.1	5.1 ± 0.3
HX-NBR-L40	3.9 ± 0.2	433.6 ± 74	1.6 ± 0.1	2.3 ± 0.1

**Table 4:** T05%, T50%, and char residue values of XNBR/lignin composite rubbers with three distinct carboxyl contents.

Samples	T05% (℃)	T50% (℃)	Char Residue Values (%)
NBR	345.3	440.3	10.3
NBR-L10	322.3	439.2	11.9
NBR-L20	308.2	439.7	12.6
NBR-L30	299.5	440.7	14.8
NBR-L40	282.2	441.2	17.6
IX-NBR	302.5	443.2	8.7
IX-NBR-L10	311.1	442.8	7.69
IX-NBR-L20	291.6	445.8	10.9
IX-NBR-L30	279.5	445.6	13.4
IX-NBR-L40	269.8	447.3	15.5
HX-NBR	335.1	442.3	7.5
HX-NBR-L10	310.5	443.5	9.9
HX-NBR-L20	303.1	445.5	13.0
HX-NBR-L30	291.2	447.2	15.4
HX-NBR-L40	281.6	447.7	17.9

## Data Availability

The data presented in this study are available upon request from the corresponding author.

## Supplementary Materials

The following supporting information can be downloaded at: https://www.mdpi.com/article/10.3390/polym17172332/s1, Figure S1: SEM images of NBR-L40 (a), IX-NBR-L40 (b), and HX-NBR-L40 (c) at a scale of 50 μm.

## References

[1] Ge Y., Diao P., Li X., Zhou Y., Xu Z., Bian H., Xiao Y., Wang C. (2025). Application of DPG/KH550 Modified Pyrolysis Carbon Black in Oil and High Temperature-Resistant NBR Composites. J. Appl. Polym. Sci..

[2] Pinedo B., Hadfield M., Tzanakis I., Conte M., Anand M. (2018). Thermal Analysis and Tribological Investigation on TPU and NBR Elastomers Applied to Sealing Applications. Tribol. Int..

[3] Li S., Liu T., Wang L., Wang Z. (2013). Dynamically Vulcanized Nitrile Butadiene Rubber/Ethylene-Vinyl Acetate Copolymer Blends Compatibilized by Chlorinated Polyethylene. J. Macromol. Sci. B Phys..

[4] Li Y., Wu J., Chen Z., Zhang Z., Su B., Wang Y. (2024). The Influence of Oil and Thermal Aging on the Sealing Characteristics of NBR Seals. Polymers.

[5] Wei Q., Yang D., Yu L., Ni Y., Zhang L. (2020). Fabrication of Carboxyl Nitrile Butadiene Rubber Composites with High Dielectric Constant and Thermal Conductivity Using Al2O3@PCPA@GO Hybrids. Compos. Sci. Technol..

[6] Dunn J.R., Vara R.G. (1983). Oil Resistant Elastomers for Hose Applications. Rubber Chem. Technol..

[7] Hashimoto K., Maeda A., Hosoya K., Todani Y. (1998). Specialty Elastomers for Automotive Applications. Rubber Chem. Technol..

[8] Pal K., Das T., Pal S.K., Das C.K. (2008). Use of Carboxylated Nitrile Rubber and Natural Rubber Blends as Retreading Compound for OTR Tires. Polym. Eng. Sci..

[9] Sombatsompop N., Wimolmala E., Sirisinha C. (2008). Fly Ash Particles and Precipitated Silica as Fillers in Rubbers. III. Cure Characteristics and Mechanical and Oil-Resistance Properties of Acrylonitrile-Butadiene Rubber. J. Appl. Polym. Sci..

[10] Zhang Z.F., Liu X.T., Yang K., Zhao S.G. (2019). Design of Coordination-Crosslinked Nitrile Rubber with Self-Healing and Reprocessing Ability. Macromol. Res..

[11] Pal K., Pal S.K., Das C.K., Kim J.K. (2011). Effect of Fillers on Morphological Properties and Wear Characteristics of XNBR/NR Blends. J. Appl. Polym. Sci..

[12] Severe G., White J.L. (2005). Dynamically Vulcanized Blends of Oil-Resistant Elastomers with HNBR. J. Appl. Polym. Sci..

[13] Chakraborty S.K., De S.K. (1982). Epoxy-Resin-Cured Carboxylated Nitrile Rubber. J. Appl. Polym. Sci..

[14] Brown H.P. (1963). Crosslinking Reactions of Carboxylic Elastomers. Rubber Chem. Technol..

[15] Xu C., Wu W., Nie J., Fu L., Lin B. (2019). Preparation of Carboxylic Styrene Butadiene Rubber/Chitosan Composites with Dense Supramolecular Network via Solution Mixing Process. Compos. Part A: Appl. Sci. Manuf..

[16] Paran S.M.R., Naderi G., Ghoreishy M.H.R. (2016). XNBR-Grafted Halloysite Nanotube Core-Shell as a Potential Compatibilizer for Immiscible Polymer Systems. Appl. Surf. Sci..

[17] Cheng C., Chen Z., Huang Z., Zhang C., Tusiime R., Zhou J., Sun Z., Liu Y., Yu M., Zhang H. (2020). Simultaneously Improving Mode I and Mode II Fracture Toughness of the Carbon Fiber/Epoxy Composite Laminates via Interleaved with Uniformly Aligned PES Fiber Webs. Compos. Part A: Appl. Sci. Manuf..

[18] Laskowska A., Zaborski M., Boiteux G., Gain O., Marzec A., Maniukiewicz W. (2014). Ionic Elastomers Based on Carboxylated Nitrile Rubber (XNBR) and Magnesium Aluminum Layered Double Hydroxide (Hydrotalcite). Express Polym. Lett..

[19] Sahoo S., Bhowmick A.K. (2007). Influence of ZnO Nanoparticles on the Cure Characteristics and Mechanical Properties of Carboxylated Nitrile Rubber. J. Appl. Polym. Sci..

[20] Zainal Abidin Z., Mamauod S.N.L., Romli A.Z., Sarkawi S.S., Zainal N.H. (2023). Synergistic Effect of Partial Replacement of Carbon Black by Palm Kernel Shell Biochar in Carboxylated Nitrile Butadiene Rubber Composites. Polymers.

[21] Yang S., Liang P., Hua K., Peng X., Zhou Y., Cai Z. (2018). Preparation of Carboxylated Nitrile Butadiene Rubber/Fly Ash Composites by in-Situ Carboxylate Reaction. Compos. Sci. Technol..

[22] Szadkowski B., Marzec A., Zaborski M. (2019). Effect of in Situ Silanization of Multiwalled Carbon Nanotubes on the Properties of NBR/MWCNT-OH Composites. Polym. Plast. Technol. Eng..

[23] Pingot M., Szadkowski B., Zaborski M. (2018). Effect of Carbon Nanofibers on Mechanical and Electrical Behaviors of Acrylonitrile-butadiene Rubber Composites. Polym. Adv. Technol..

[24] Ha C.S. (2005). Carboxylated Nitrile Elastomer/Filler Nanocomposite: Effect of Silica Nanofiller in Thermal, Dynamic Mechanical Behavior, and Interfacial Adhesion. Macromol. Res..

[25] Ragauskas A.J., Beckham G.T., Biddy M.J., Chandra R., Chen F., Davis M.F., Davison B.H., Dixon R.A., Gilna P., Keller M. (2014). Lignin Valorization: Improving Lignin Processing in the Biorefinery. Science.

[26] Liu X., Zhou X., Yang C., Haung J., Wang P. (2022). Effect of interfacial interaction between Nano-SiO2 and NBR on tribological properties of NBR water-lubricated bearings. Wear.

[27] Collins M.N., Nechifor M., Tanasă F., Zănoagă M., McLoughlin A., Stróżyk M.A., Culebras M., Teacă C.-A. (2019). Valorization of Lignin in Polymer and Composite Systems for Advanced Engineering Applications—A Review. Int. J. Biol. Macromol..

[28] Xia Z., Li J., Zhang J., Zhang X., Zheng X., Zhang J. (2020). Processing and Valorization of Cellulose, Lignin and Lignocellulose Using Ionic Liquids. J. Bioresour. Bioprod..

[29] Lizundia E., Sipponen M.H., Greca L.G., Balakshin M., Tardy B.L., Rojas O.J., Puglia D. (2021). Multifunctional Lignin-Based Nanocomposites and Nanohybrids. Green Chem..

[30] Barana D., Ali S.D., Salanti A., Orlandi M., Castellani L., Hanel T., Zoia L. (2016). Influence of Lignin Features on Thermal Stability and Mechanical Properties of Natural Rubber Compounds. ACS Sustain. Chem. Eng..

[31] Parvathy G., Sethulekshmi A.S., Jayan J.S., Raman A., Saritha A. (2021). Lignin Based Nano-Composites: Synthesis and Applications. Process Saf. Environ. Prot..

[32] Liu Z.-H., Li B.-Z., Yuan J.S., Yuan Y.-J. (2022). Creative Biological Lignin Conversion Routes toward Lignin Valorization. Trends Biotechnol..

[33] Zhou Y., Fan M., Chen L., Zhuang J. (2015). Lignocellulosic Fibre Mediated Rubber Composites: An Overview. Compos. Part B Eng..

[34] Chang B.P., Gupta A., Muthuraj R., Mekonnen T.H. (2021). Bioresourced Fillers for Rubber Composite Sustainability: Current Development and Future Opportunities. Green Chem..

[35] Burfield D.R., Lim K., Law K. (1984). Epoxidation of Natural Rubber Latices: Methods of Preparation and Properties of Modified Rubbers. J. Appl. Polym. Sci..

[36] Qiu J., Yuan S., Xiao H., Liu J., Shen T., Tan Z., Zhuang W., Ying H., Li M., Zhu C. (2023). Study on Lignin Amination for Lignin/SiO2 Nano-Hybrids towards Sustainable Natural Rubber Composites. Int. J. Biol. Macromol..

[37] Mei J., Liu W., Huang J., Qiu X. (2019). Lignin-Reinforced Ethylene-Propylene-Diene Copolymer Elastomer via Hydrogen Bonding Interactions. Macromol. Mater. Eng..

[38] Roy K., Debnath S.C., Pongwisuthiruchte A., Potiyaraj P. (2021). Recent Advances of Natural Fibers Based Green Rubber Composites: Properties, Current Status, and Future Perspectives. J. Appl. Polym. Sci..

[39] Campos G.N., Rocha E.B.D., Furtado C.R.G., Figueiredo M.A.G., Sousa A.M.F. (2024). Using carboxyl groups to improve the compatibility of XNBR/lignin composites. Polym. Compos..

[40] (2009). Vulcanized rubbers or thermoplastics—Determination of tensile stress-strain proper-ties.

[41] Kantala C., Wimolmala E., Sirisinha C., Sombatsompop N. (2009). Reinforcement of Compatibilized NR/NBR Blends by Fly Ash Particles and Precipitated Silica. Polym. Adv. Technol..

[42] Gregorová A., Košíková B., Moravčík R. (2006). Stabilization Effect of Lignin in Natural Rubber. Polym. Degrad. Stabil..

[43] Paran S.M.R., Naderi G., Mosallanezhad H., Movahedifar E., Formela K., Saeb M.R. (2020). Microstructure and Mechanical Properties of Carboxylated Nitrile Butadiene Rubber/Epoxy/XNBR-Grafted Halloysite Nanotubes Nanocomposites. Polymers.

[44] Mousa A., Heinrich G., Wagenknecht U. (2011). Cure Characteristics and Mechanical Properties of Carboxylated Nitrile Butadiene Rubber (XNBR) Vulcanizate Reinforced by Organic Filler. Polym. Plast. Technol. Mater..

[45] Mohamad Aini N.A., Othman N., Hussin M.H., Sahakaro K., Hayeemasae N. (2020). Lignin as Alternative Reinforcing Filler in the Rubber Industry: A Review. Front. Mater..

[46] Ferruti F., Carnevale M., Giannini L., Guerra S., Tadiello L., Orlandi M., Zoia L. (2024). Mechanochemical Methacrylation of Lignin for Biobased Reinforcing Filler in Rubber Compounds. ACS Sustain. Chem. Eng..

[47] Thakur V.K., Thakur M.K., Raghavan P., Kessler M.R. (2014). Progress in Green Polymer Composites from Lignin for Multifunctional Applications: A Review. ACS Sustainable Chem. Eng..

[48] Barana D., Orlandi M., Zoia L., Castellani L., Hanel T., Bolck C., Gosselink R. (2018). Lignin Based Functional Additives for Natural Rubber. ACS Sustain. Chem. Eng..

[49] Shi X., Sun S., Zhao A., Zhang H., Zuo M., Song Y., Zheng Q. (2021). Influence of Carbon Black on the Payne Effect of Filled Natural Rubber Compounds. Compos. Sci. Technol..

[50] Wu Z., Lin X., Teng J., Li M., Song J., Huang C., Wang R., Ying H., Zhang L., Zhu C. (2023). Recent Advances of Lignin Functionalization for High-Performance and Advanced Functional Rubber Composites. Biomacromolecules.

[51] Kruželák J., Džuganová M., Hložeková K., Kvasničáková A., Ház A., Nadányi R., Krump H., Hudec I. (2024). Sulfur and Peroxide Curing of NBR Based Rubber Compounds Filled with Kraft Lignin and Calcium Lignosulfonate. J. Appl. Polym. Sci..

[52] Jang G.G., Nguyen N.A., Bowland C.C., Ho H.C., Keum J.K., Naskar A.K. (2020). Effects of Graphene Surface Functionalities towards Controlled Reinforcement of a Lignin Based Renewable Thermoplastic Rubber. Compos. Sci. Technol..

[53] Košíková B., Gregorová A., Osvald A., Krajčovičová J. (2007). Role of Lignin Filler in Stabilization of Natural Rubber–Based Composites. J. Appl. Polym. Sci..

[54] Datta J., Parcheta P., Surówka J. (2017). Softwood-Lignin/Natural Rubber Composites Containing Novel Plasticizing Agent: Preparation and Characterization. Ind. Crops Prod..

