# Supplementary material for: Effect of Carboxyl Content on Mechanical Properties of Lignin/Carboxylated Nitrile Rubber Compounds
Source: Polymers (Basel). 2025 Aug 28;17(17):2332. doi: 10.3390/polym17172332 (PMC12431394; doi:10.3390/polym17172332)
Supplement: Supplementary file 1 [file polymers-17-02332-s001.zip › polymers-3786646-supplementary.pdf]

Supporting Information

Effect of Carboxyl Content on Mechanical Properties of Lignin/Carboxylated Nitrile Rubber Compounds

Hongbing Zheng and Dongmei Yue*

Beijing University of Chemical Technology, Beijing 100029, PR China; Key Laboratory of Beijing City on Preparation and Processing of Novel Polymer Materials, Beijing 100029, P.R. China; zhenghongbing@petrochina.com.cn

*Correspondence: yuedm@mail.buct.edu.cn

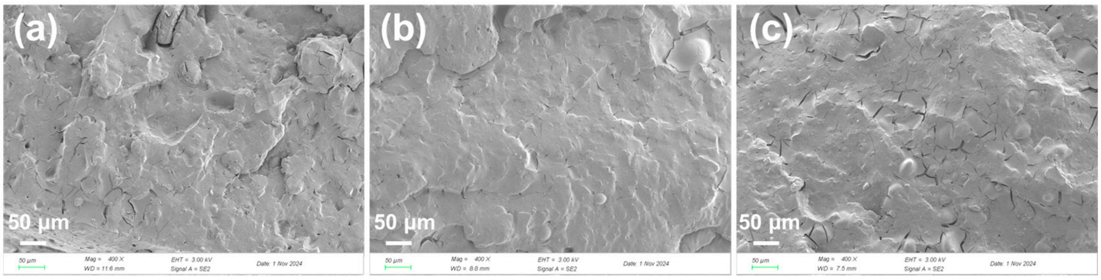

Figure S1. SEM images of NBR-L40 (a), IX-NBR-L40 (b), and HX-NBR-L40 (c) at a scale of 50 μm.

。
